# Effects of sub-atmospheric pressure and dissolved oxygen concentration on lesions generated in ex vivo tissues by high intensity focused ultrasound

**DOI:** 10.1186/s12938-021-00926-z

**Published:** 2021-09-15

**Authors:** Min He, Zhiqiang Zhong, Deping Zeng, Xiaobo Gong, Zhibiao Wang, Faqi Li

**Affiliations:** 1 State Key Laboratory of Ultrasound in Medicine and Engineering, College of Biomedical Engineering, Chongqing Medical University, Chongqing, 400016 China; 2 Chongqing Key Laboratory of Biomedical Engineering, Chongqing Medical University, Chongqing, 400016 China; 3 National Engineering Research Center of Ultrasound Medicine, Chongqing, 401121 People’s Republic of China

**Keywords:** HIFU, Cavitation, Lesion, Sub-atmospheric pressure, Dissolved oxygen concentration

## Abstract

**Background:**

Acoustic cavitation plays an important role in the medical treatment using high-intensity focused ultrasound (HIFU), but unnecessarily strong cavitation also could deform the morphology and enlarge the size of lesions. It is known that the increase of ambient hydrostatic pressure (*P*_stat_) can control the acoustic cavitation. But the question of how the decrease of *P*_stat_ and dissolved oxygen concentration (DOC) influence the strength of cavitation has not been thoroughly answered. In this study, we aimed to investigate the relationship among the *P*_stat_, DOC and the strength of cavitation.

**Methods:**

Ex vivo bovine liver tissues were immersed in degassed water with different DOC of 1.0 mg/L, 1.5 mg/L and 2.0 mg/L. Ultrasound (US) of 1 MHz and the spatial and temporal average intensity (*I*_sata_) of 6500 W/cm^2^ was used to expose two groups of in vitro bovine livers for 2 s; one group was under atmospheric pressure (*P*_stat_ = 1 bar) and the other was under sub-atmospheric pressure (*P*_stat_ = 0.1 bar). Acoustic cavitation was detected by a passive cavitation detector (PCD) during the exposure process. Echo signals at the focal zone of HIFU were monitored by B-mode ultrasound imaging before and after exposure. The difference between two pressure groups was tested using paired sample *t*-test. The difference among different DOC groups was evaluated by one-way analysis of variance (ANOVA).

**Results:**

The results demonstrated a significant difference of broadband acoustic emissions from the cavitation bubbles, echo signals on B-mode image, morphology of lesions under various conditions of ambient pressure and DOC. The lesion volume in tissue was increased with the increase of ambient pressure and DOC.

**Conclusion:**

Cavitation could be suppressed through sub-atmospheric pressure and low DOC level in liver tissue, which could provide a method of controlling cavitation in HIFU treatment to avoid unpredictable lesions.

## Background

As a noninvasive therapy for cancer treatment, high-intensity focused ultrasound (HIFU) has recently been receiving more and more attention [1–4]. The absorption of highly localized ultrasound energy by the targeted tissue in the focal region allows the temperature in situ rapidly rise over 60 °C, consequently the irreversible coagulation necrosis takes place instantaneously [5–7], while the surrounding tissues are spared from the significant damage [8]. During HIFU treatment, acoustic cavitation plays an important role through the collapse of oscillating microbubbles in tissues [9, 10]. Cavitation enhances lesion formation mainly by local high-intensity acoustic wave, thermal deposition of acoustic radiation from the compressed bubbles and the viscous loss of bubble oscillation through tissue organization and the body liquid [11–13]. Coussios et al. [14] indicated that the appearance of cavitation bubbles would change the acoustic impedance and attenuation coefficient, resulting in more severe lesion. Chen et al. [15] and Watkin et al. [16] found that the morphology and size of targeted tissue were out of control due to severe cavitation. Sokka et al. [17] observed that lesions with cavitation in the region closer to the transducer of thigh tissue of rabbit were about 2 ~ 3 times larger by volume than the lesions under the same exposure conditions without cavitation. Hynynen [18] showed that an enhanced heating effect in dog’s thigh tissue during sonications, and concluded that such effect should be avoided in clinical therapy because they might lead to unpredictable thermal and mechanical damage. Chapelon et al*.* [19–21] observed irregular lesion outside the focal zone when uncontrolled cavitation occurred during HIFU treatment, which affected the therapeutic effects of targeted tissue. They strongly recommended that acoustic cavitation during the treatment should be avoided.

Cyril et al*.* [22] showed that using gated sonication instead of continuous sonications can remove residual cavitation nuclei between pulses with gated sonications to weaken the cavitation. Several researchers elevated the ambient hydrostatic pressure (*P*_stat_) to suppress cavitation [23–25] in continuous HIFU treatment. He et al*.* [26] proved that elevating the ambient hydrostatic pressure can restrain the acoustic cavitation, the morphology of lesion was regular and the size was smaller under higher *P*_stat_. Caupin and Herbert [27] demonstrated that cavitation threshold would increase with the increase of hydrostatic pressure, but sub-atmospheric pressure would strengthen the process of expansion motion of microbubbles and promote the activities of cavitation bubbles. After systematic experiment and molecular dynamics simulations, Kinjo and Matsumoto [28] manifested that cavitation nuclei would appear immediately under the condition of a sub-atmospheric pressure of 0.2 bar, and the appearance time would be delayed when under sub-atmospheric pressure with *P*_stat_ equal to 0.4 bar. However, all these studies were all performed in water. It is still unknown whether the sub-atmospheric pressure would influence cavitation activities in biological tissues such as the liver during the process of HIFU exposure.

Dissolved oxygen concentration (DOC) also plays a significant role on the cavitation bubble during HIFU treatment. A change in the gas content of a medium can affect the propagation of focused ultrasound beam, leading to an unpredictable lesion. Saito and Soetanto [29] showed that the quantity of microbubbles would increase with the increase of DOC during ultrasound irradiation. Tuziuti et al. [30] indicated that cavitation was more active as the increase of DOC in a solution. Stomach and intestinal preparation including fasting and water-deprivation were necessary before the clinical treatment of liver cancer, pancreatic cancer and kidney cancer [31–33]. When treating prostate cancer and uterine fibroid, and bladder injection with degassed water as acoustic coupling medium through the catheter was also needed [34, 35], and the DOC of degassed water should be less than 4 mg/L, according to the treatment standard issued by the State Food & Drug Administration of China [36]. In HIFU experiments, isolated biological tissues such as bovine liver were generally degassed for 1 h [37]. However, the value of DOC in bovine liver tissue was not clearly stated in literature.

Based on these findings, we hypothesized that *P*_stat_ and DOC can control the cavitation in tissue during HIFU treatment. Thus, in the present study, we examined whether *P*_stat_ and DOC can regulate the lesion morphology and smaller the lesion size induced by cavitation in HIFU treatment. And if so, to analyze the mechanism of lesion formation and cavitation behavior in ex vivo bovine livers.

## Results

### DOC in degassed water and inside the tissue

Table 1 shows the DOC of degassed water and inside the tissue. Under the three levels of DOC (1.0 mg/L, 1.5 mg/L, 2.0 mg/L), there is no significant difference between the DOC in degassed water and inside the water. Because of the DOC test inside the tissue took much more time than the test in water, we used the DOC in water to present the DOC inside the tissue.

**Table 1:** The comparison of DOC in degassed water and inside the tissue

Dissolved oxygen concentration (mg/L)	Test in degassed water (mg/L)	Mean value in degassed water (mg/L)	Test in tissue (mg/L)	Mean value in tissue (mg/L)	Rate of deviation between water and tissue (%)
1.0	1.07	1.04	0.95	1.05	0.77
	0.99		1.06		
	1.05		1.03		
	0.98		1.10		
	1.10		1.08		
1.5	1.48	1.52	1.53	1.54	1.45
	1.40		1.57		
	1.52		1.46		
	1.60		1.61		
	1.58		1.52		
2.0	1.98	2.02	2.10	2.03	0.40
	2.06		2.03		
	2.00		1.95		
	1.98		2.09		
	2.09		1.98		

### Cavitation signal

**Fig. 1:**
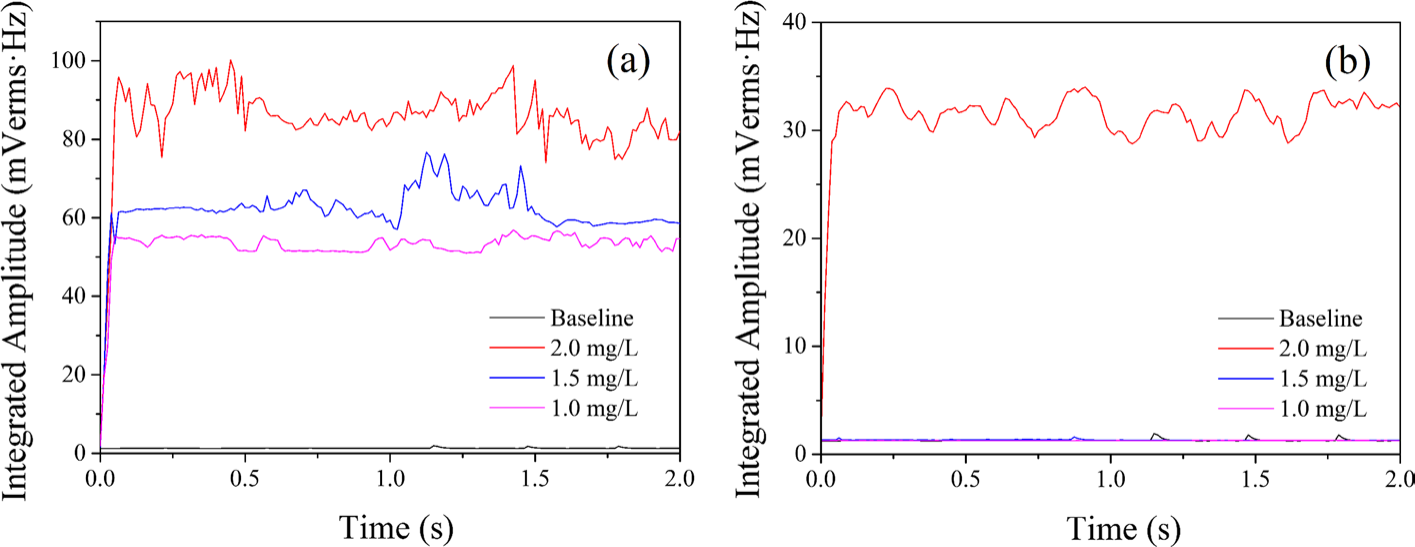
Broadband noise during HIFU exposure: **a** atmospheric pressure, **b** sub-atmospheric pressure. The black solid line was the baseline detected without HIFU exposure. The red, blue and purple solid lines represent the broadband noise signal at the DOC of 2.0 mg/L, 1.5 mg/L and 1.0 mg/L, respectively

Under the conditions of atmospheric pressure and sub-atmospheric pressure, the broadband emissions from ex vivo bovine liver exposed by HIFU in degassed water with different DOC are shown in Fig. 1. The black solid line was the baseline which was detected without HIFU exposure. The signals of broadband emissions under atmospheric pressure (Fig. 1a) were higher than that under sub-atmospheric pressure (Fig. 1b) when DOC was the same. Under the condition of atmospheric pressure (1 bar), the signal of broadband emissions at three DOC levels (1.0 mg/L, 1.5 mg/L and 2.0 mg/L) were all above the noise level of the system during HIFU exposure. Moreover, the signal of broadband noise increased with an increase of the DOC levels. Under the condition of sub-atmospheric pressure (0.1 bar), there was no signal of broadband emissions above the noise level of the system except under DOC of 2.0 mg/L. The violent collapse of transient cavitation is the only source of broadband emissions [15], these results indicated that each DOC level had transient cavitation behavior during HIFU exposure under atmospheric pressure, but under the condition of sub-atmospheric pressure, only 2.0 mg/L had transient cavitation behavior.

### Gray-level variation on B-mode ultrasound

**Fig. 2:**
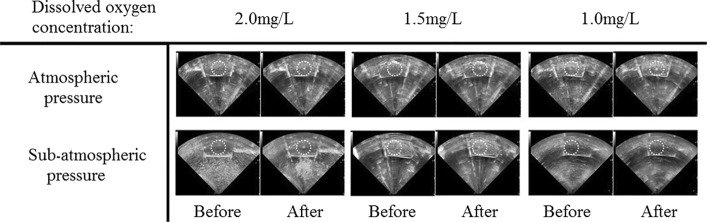
B-mode ultrasound image before and after HIFU exposure. The white dotted circle flagged the HIFU focus zone. Hyper-echoic occurred under all conditions except at sub-atmospheric pressure with the DOC of 1.0 mg/L

Figure 2 shows B-mode ultrasound image change before and after HIFU exposure. HIFU focus was located in the center of white dotted circle during the exposure. Under the condition of atmospheric pressure (1 bar), hyper-echoic change was found in the focus after HIFU exposure under the condition of different DOC levels. Under sub-atmospheric pressure (0.1 bar), hyper-echoic changes were observed found under DOC conditions of 1.5 mg/L and 2.0 mg/L, but no hyper-echo at 1.0 mg/L DOC.

### *Lesions on *ex vivo* bovine livers*

**Fig. 3:**
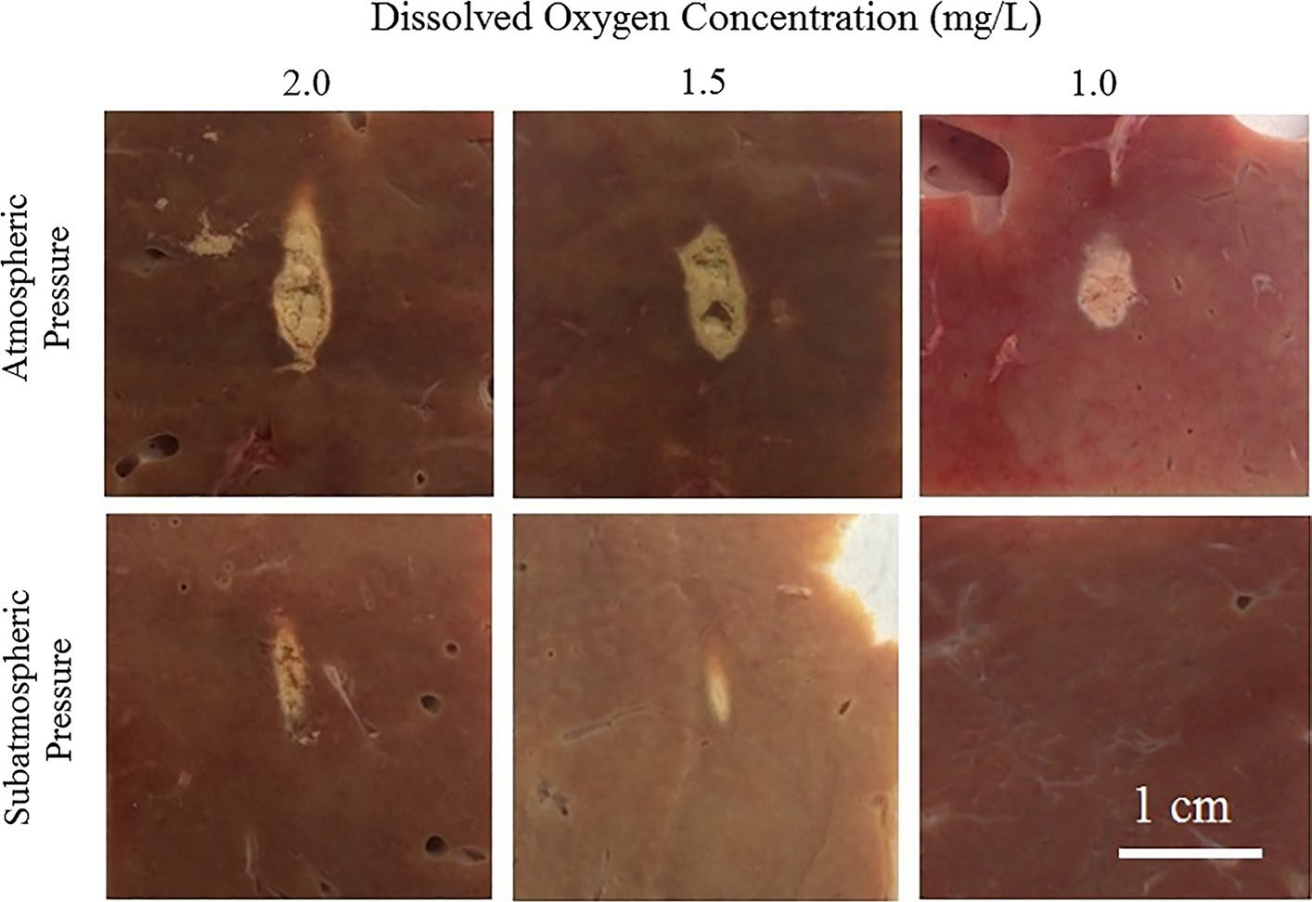
Morphology variation of lesions in ex vivo bovine liver. The first row was experimented under the condition of atmospheric pressure, and the second row was experimented under the condition of sub-atmospheric pressure. The column represented the HIFU exposure conducted under the DOC of 2.0 mg/L, 1.5 mg/L and 1.0 mg/L. Lesion appeared under all conditions except at sub-atmospheric pressure with the DOC of 1.0 mg/L

The morphology of lesions in ex vivo bovine liver exposed by HIFU is shown in Fig. 3. Under atmospheric pressure (1 bar), the morphology of lesions was very different from each other. In the 2.0 mg/L DOC group, the lesion was teardrop-shaped. In the 1.5 mg/L and 1.0 mg/L DOC groups, the lesions were approximately ellipsoidal. Under sub-atmospheric pressure (0.1 bar), in the 2.0 mg/L DOC group, the lesion was approximately cigar-shaped. In the 1.5 mg/L DOC group, the lesion was also cigar-shaped, but the size of lesion was smaller than that in the 2.0 mg/L group, with homogeneous coagulation necrosis in the central part. In the 1.0 mg/L DOC group there was no significant lesion formed in the liver tissue. In addition, when the DOC level was the same, the size of lesions at atmospheric pressure (1 bar) were larger than that at sub-atmospheric pressure (0.1 bar), and damage on the central zone of lesion at atmospheric pressure (1 bar) was severer than that at sub-atmospheric pressure (0.1 bar).

**Fig. 4:**
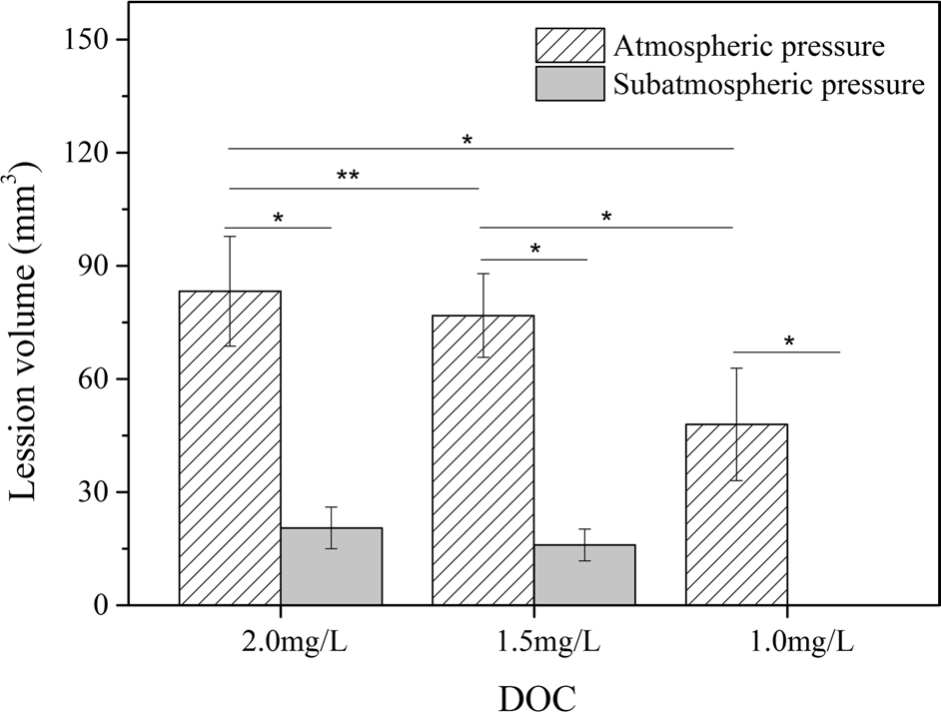
Lesion volume variation in ex vivo bovine liver after HIFU exposure. At atmospheric pressure (1 bar), the volume of lesions in the 2.0 mg/L and 1.5 mg/L DOC group were 83.28 ± 14.56 mm^3^ and 76.84 ± 11.07 mm^3^, respectively. At sub-atmospheric pressure (0.1 bar), the volume of lesions in the 2.0 mg/L, 1.5 mg/L and 1.0 mg/L groups were 20.53 ± 5.54 mm^3^, 16.01 ± 4.22 mm^3^ and 0.00 ± 0.00 mm^3^, respectively. Lesion volume under atmospheric pressure was significantly different from that under sub-atmospheric pressure when DOC was kept the same. *Represents *p* < 0.05 for significance of difference between the two groups, **represents *p* > 0.05 for no significance of difference between the two groups

Figure 4 shows the volume of lesions in ex vivo bovine liver exposed by HIFU under atmospheric pressure (1 bar) and sub-atmospheric pressure (0.1 bar) with different DOC conditions. At atmospheric pressure (1 bar), the volume of lesions in the 2.0 mg/L and 1.5 mg/L DOC group were 83.28 ± 14.56 mm^3^ and 76.84 ± 11.07 mm^3^, respectively. There was no significant difference between two groups (*p* > 0.05). However, when DOC was equal to 1.0 mg/L, the volume of lesion was 47.98 ± 14.92 mm^3^, which was significantly decreased while compared with the 2.0 mg/L and 1.5 mg/L groups (*p* < 0.05). At sub-atmospheric pressure (0.1 bar), the volume of lesions in the 2.0 mg/L, 1.5 mg/L and 1.0 mg/L groups were 20.53 ± 5.54 mm^3^, 16.01 ± 4.22 mm^3^ and 0.00 ± 0.00 mm^3^, respectively. There were significant differences between any two (*p* < 0.05), indicating that the volume of lesion was increased with the increase of DOC levels at either atmospheric pressure or sub-atmospheric pressure. Meanwhile, the volume of lesion in tissue at atmospheric pressure (1 bar) was larger than that at sub-atmospheric pressure (0.1 bar) while DOC level kept the same (*p* < 0.05).

## Discussion

The main mechanisms of coagulation necrosis induced by HIFU are thermal deposition and cavitation effect [38]. During the process of HIFU exposure, the absorption of sound energy can result in thermal deposition in tissue, leading to formation of lesions [6]. Moreover, cavitation can accelerate the rise of the in situ temperature and enlarge the size of lesions [18]. Cavitation includes stable cavitation and transient cavitation [39], and the accelerated temperature increasing during HIFU exposure comes from transient cavitation [17].

Overpressure has been proved to suppress cavitation and has been used to study the influence of cavitation on HIFU-induced lesions [24–26]. This study investigates the effect of atmospheric pressure and sub-atmospheric pressure on the formation of the lesions. Under various DOC conditions (2.0 mg/L, 1.5 mg/L or 1.0 mg/L), the broadband noise at sub-atmospheric pressure (0.1 bar) is significantly lower than that at atmospheric pressure (1 bar). It manifests that sub-atmospheric pressure can partly suppress cavitation in liver tissue during HIFU exposure, which is different from the report that sub-atmospheric pressure can strengthen cavitation activity in water by Caupin and Herbert [27]. We think that although sub-atmospheric pressure can enhance the expansion process of cavitation bubble, the mechanical strain of sub-atmospheric pressure can also disperse the bubble distribution in tissue, resulting in that cavitation bubble cannot stably form and concentrate in the focus and the signal of sound scattering in focus is distinctly weakened. This is demonstrated by our bovine liver experiments at sub-atmospheric pressure, which reveals that the volume of the lesions is smaller than that at atmospheric pressure (*p* < 0.05). Under the experiment parameter under which cavitation occurred, the center of lesions appears to be mechanically damaged due to the collapse of cavitation bubbles. Without cavitation occurring, the lesion undergoes homogeneous necrosis, and there is no lesion formed in bovine liver tissue while DOC is equal to 1.0 mg/L.

This study also demonstrates a certain quantity of cavitation nuclei is needed for the occurrence of cavitation. Under atmospheric pressure, PCD results showed that cavitation activities exist during HIFU exposure under the condition of three DOC levels, with the hyper-echoic changed observed on B-mode ultrasound after HIFU exposure. With the increase of DOC level, the strength of the cavitation signal enhances gradually during the process of exposure, leading to the increased volume of the lesion in bovine liver tissue. However, under sub-atmospheric pressure, cavitation signal only appeared in 2.0 mg/L DOC level group, and the B-mode ultrasound image without hyper-echoic occurred only at the DOC 1.0 mg/L group. The volume of the lesion at the condition of 2.0 mg/L DOC is 20.53 ± 5.54 mm^3^, which is larger than the lesion volume of 16.01 ± 4.22 mm^3^ at the DOC 1.5 mg/L group (*p* < 0.05), but no lesion is observed at the DOC 1.0 mg/L group, indicating that lower DOC level could produce less gas content in water, and less than the critical number of cavitation nuclei that makes cavitation difficult occur during HIFU exposure. On the contrary, an increase of gas content in both water and tissues could facilitate the occurrence of cavitation and lesion formation, which could improve the efficiency of HIFU treatment through microbubbles [40]. In addition, Rabkin et al*.* [41] reported that hyper-echo on B-mode ultrasound was produced by boiling bubbles of water during HIFU exposure. But the boiling point of water at sub-atmospheric pressure (0.1 bar) is only 56 °C, which is much lower than that at atmospheric pressure (1 bar). This might help understand our findings that under the condition of sub-atmospheric pressure and DOC 1.5 mg/L level, PCD showed no cavitation during HIFU exposure but a significant hyper-echo on B-mode ultrasound and homogenous lesion in bovine liver tissue.

## Conclusions

Using ex vivo bovine livers, we investigated the effect of *P*_stat_ and DOC levels on the formation of HIFU-induced lesions in this study. Our results showed that the occurrence of cavitation could be suppressed through sub-atmospheric pressure and low DOC level in liver tissue. This could provide a method of controlling cavitation in HIFU treatment which could avoid unpredictable lesions. Conversely, coagulation necrosis could be enhanced by increasing the quantity of cavitation nuclei in tissues.

## Methods

**Fig. 5:**
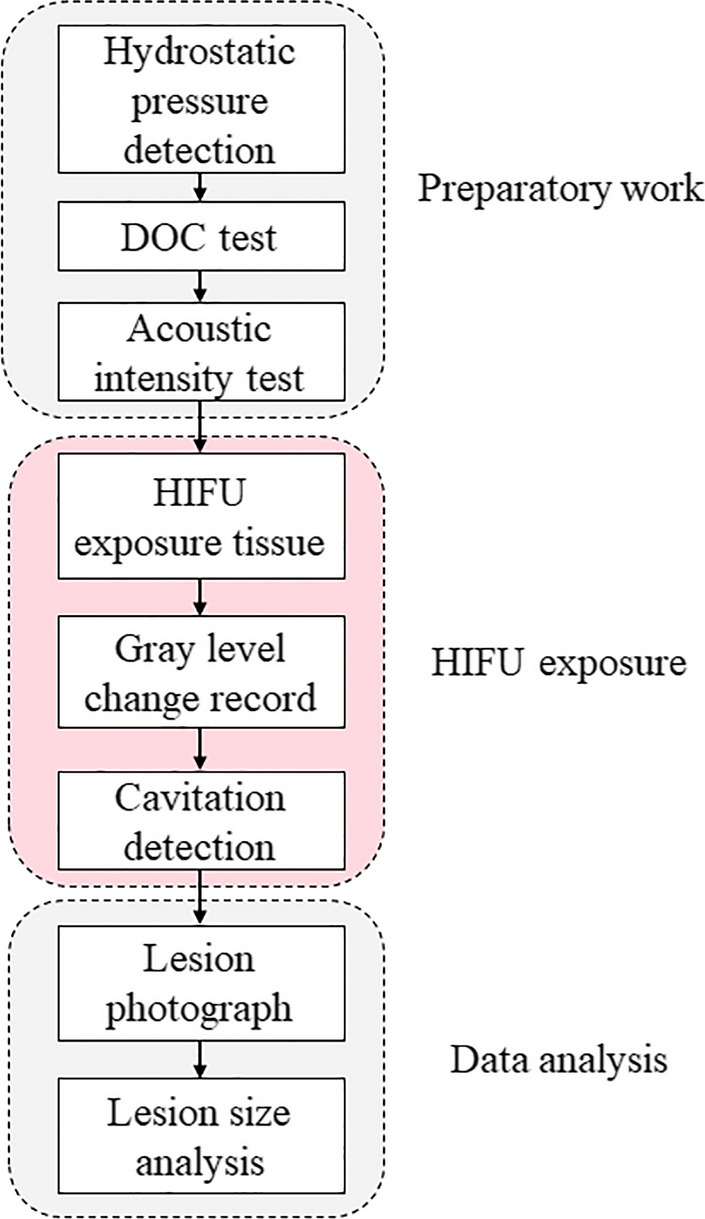
The flowchart of methods

As shown in Fig. 5, the experiment is mainly composed of three parts: the preparatory work, the HIFU exposure and the data analysis. The preparatory work and the HIFU exposure experiment were performed in a stainless chamber (50 cm *L* × 50 cm W × 70 cm *H*) which was filled with degassed water. The data analysis was carried out on the computer.

### Experimental equipment

**Fig. 6:**
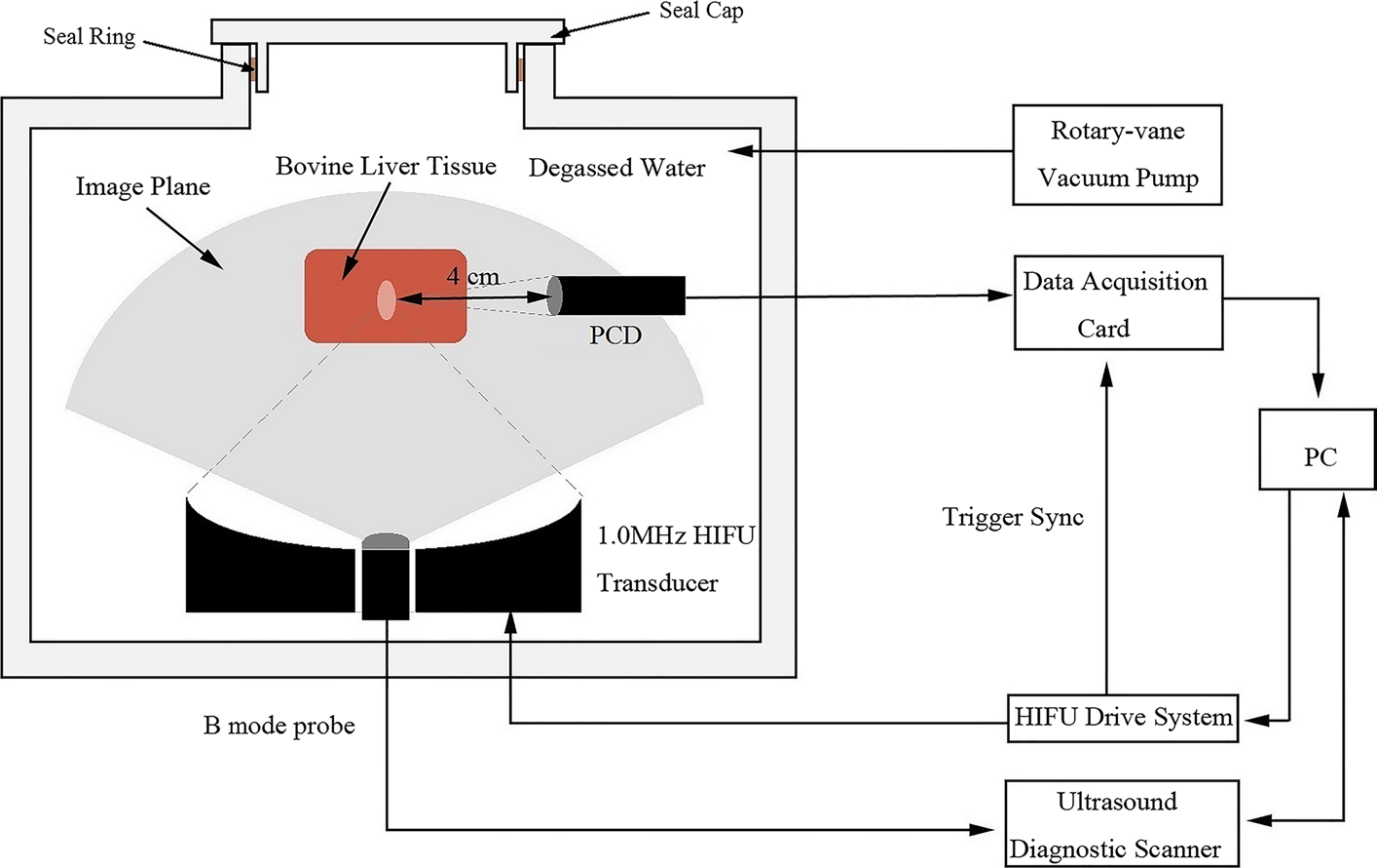
Schematic diagram of the experimental system. The HIFU transducer was driven by the HIFU drive system. The input power was set on the PC. The PCD signal was acquired by the data acquisition card and shown on the PC. The ultrasound diagnostic scanner, which was controlled by PC, was used to scan the B-mode image of tissue

As shown in Fig. 6, the major experimental system (Jisheng 1#, Chongqing Haifu Technology Co., Ltd, Chongqing, China) includes a 1.0 MHz HIFU transducer (12–32, Chongqing Haifu Technology Co., Ltd, Chongqing, China), with an aperture diameter of 220 mm and a focal length of 170 mm. The probe of the B-mode ultrasound (PA230E, ESAOTE Co., Genova, Italy) was mounted in the central hole of HIFU transducer, sharing the same focus with HIFU transducer, so that its imaging plane intersected the HIFU axis along its axial length. Data cables connected to a computer through the hermetic feedthrough. A HIFU drive system, a water processing system, which can prepare for degassed water of different DOC, and sample holder are all provided by Chongqing Haifu (HIFU) Technology Co., Ltd. A rotary vane vacuum pump (2XZ-2, Taizhou Qiushi vacuum pump Co., Ltd, Zhejiang, China) was used to control the condition of *P*_stat_ in the chamber. DOC in water and tissue was monitored by a portable dissolved oxygen meter (LDOTM 550A-12, YSI Co., Yellow Springs, OH, USA), with measurement range of 0 ~ 50 mg/L and resolution of 0.01 mg/L.

### Dissolved oxygen concentration and hydrostatic pressure detection

Degassed water at different DOC levels was prepared by regulating the water flow of a water processing system. The experimental chamber was filled with degassed water at DOC of 1.0 mg/L, 1.5 mg/L and 2.0 mg/L, respectively, with the temperature kept at 37 ℃, according to the standard protocol of HIFU treatment system [36]. The degassed bovine liver specimens were mounted in the sample holder, and then placed in the experimental chamber. Ten minutes later, the DOC of degassed water and inside the tissue was detected for five times, shown in Table 1. The DOC in water was in accord with the DOC inside the tissue. Therefore, we used the DOC in water to present the DOC inside the tissue. *P*_stat_ in sealed experimental chamber was changed by a vacuum pump which was turned off after 5 min to keep the experimental chamber under the *P*_stat_ of 0.1 bar, which was the maximum negative pressure that vacuum pump could stably support.

### Acoustic intensity test

Acoustic spatial and temporal averaged intensity (*I*_sata_) was 6500 W/cm^2^ for ultrasound applied to ex vivo bovine livers, where cavitation could be detected clearly under atmospheric pressure. Before the exposure experiment, the acoustic power generated was measured by the radiation force method [42], and the sound field was scanned by needle-type hydrophone (HFO-660, ONDA, Sunnyvale, CA, USA), in order to ensure every experiment had the same acoustic output.

### HIFU exposure

The liver specimens were taken from the portion of fresh bovine liver with less connective tissues and blood vessels within 6 h after the animal was killed. It was then cut into blocks of 12 cm × 6 cm × 4 cm, soaked in 0.9% saline, and degassed by vacuum pump for 60 min. During the experiment, 1-MHz HIFU transducer was used to generate a continuous wave of exposure parameter of *I*_sata_ 6500 W/cm^2^ for 2 s to expose bovine liver specimens. Experiments were done under two *P*_stat_: atmospheric pressure (*P*_stat_ = 1 bar) and sub-atmospheric pressure (*P*_stat_ = 0.1 bar). Experiments with same exposure conditions were repeated ten times. The gray-level variation of bovine livers at HIFU focus was monitored by B-mode ultrasound before and after HIFU exposure. Moreover, the DOC of water was detected by the portable dissolved oxygen analyzer before and after each experiment. The deviation of DOC in each group was within ± 0.1 mg/L.

### Cavitation detection

A passive cavitation detection (PCD) (V309-SU, Olympus Panametrics NDT Inc, Waltham, MA, USA) with a central frequency of 5 MHz was used to detect the cavitation characteristics to compare the cavitation behavior. The broadband focusing PCD transducer with the diameter of 13 mm and focal length of 40 mm was fixed on the sample holder, shown in Fig. 6. Acoustic signal from ex vivo bovine liver was recorded by the high-speed data acquisition card with sampling rate of 20 MHz (PXie-5122, National Instruments Co., USA). The corresponding spectrum of obtained signal was analyzed via fast Fourier transform on the LabView development platform (v10.0.1, National Instruments Co., Austin, TX, USA), and the sampling step length was 15 ms. The broadband noise was calculated after band-pass filter (3 ~ 7 MHz) and band-stop filter (filter stopped the signals around harmonics ± 30 kHz), to minimize the error caused by the contributions from the HIFU fundamental, second, third and fourth harmonic frequencies arising from nonlinear sound propagation.

### Data analysis

Sections of the bovine liver specimens with their cross section perpendicular to the direction of acoustic propagation of HIFU were cut immediately after exposure to observe the lesion size and shape. The lesion was photographed digitally under every exposure condition. The *HIFU measurement* software (1.0, Chongqing Haifu Technology Co., Ltd, Chongqing, China) was used to set the scale and mark the boundary of the lesion region.

### Statistics analysis

The SPSS software (19.0, IBM, Camp Takajo, NY, USA) was used for statistical analysis. The final measurement results were expressed by mean ± standard deviation (SD). Difference between the sub-atmospheric pressure group and the atmospheric group was tested using paired sample *t*-test. Statistical relationships among the three DOC groups were evaluated by one-way analysis of variance (ANOVA), and Tukey’s test was used for pairwise multiple comparisons if one-way ANOVA revealed statistical significance. A value of *p* < 0.05 was accepted as statistically significant.

## Data Availability

The datasets used and/or analyzed during the current study are available from the corresponding author on reasonable request.

## Abbreviations

DOC: Dissolved oxygen concentration;
HIFU: High-intensity focused ultrasound;
PCD: Passive cavitation detection;
*P*_stat_: Ambient hydrostatic pressure
